# Immunosuppression in stem cell clinical trials of neural and retinal cell types: A systematic review

**DOI:** 10.1371/journal.pone.0304073

**Published:** 2024-07-05

**Authors:** Shravan Gowrishankar, Matthew E. Smith, Nathan Creber, Jameel Muzaffar, Daniele Borsetto

**Affiliations:** 1 Department of ENT, Cambridge University Hospitals, Cambridge, England, United Kingdom; 2 Department of Clinical Neurosciences, University of Cambridge, Cambridge, England, United Kingdom; 3 Royal Prince Alfred Hospital, Sydney, Australia; TotiCell Limited, Bangladesh, BANGLADESH

## Abstract

**Background:**

Pharmacologic immunosuppression regimes are commonly employed in stem cell clinical trials to mitigate host immune rejection and promote survival and viability of transplanted cells. Immunosuppression and cell survival has been extensively studied in retinal and spinal tissues. The applicability of stem cell therapy is rapidly expanding to other sensory organs such as the ear and hearing. As regenerative therapy is directed to new areas, a greater understanding of immunosuppression strategies and their efficacy is required to facilitate translation to organ-specific biologic microenvironments.

**Objective:**

This systematic review appraises the current literature regarding immunosuppression strategies employed in stem cell trials of retinal and neural cells.

**Methods:**

This systematic review was performed in line with Preferred Reporting Items for Systematic Reviews and Meta-Analyses guidelines. Inclusion criteria included studies presenting data on neural or retinal cells as part of an in-human clinical trial that detailed the immunosuppression regime used. Exclusion criteria included non-English language studies, animal studies, review articles, case reports, editorials, and letters. The databases Medline, Embase, Scopus, Web of Science, and the Cochrane Library were searched from inception to February 2024. Risk of bias was evaluated using the ROBINS-I tool.

**Results:**

Eighteen articles fit the inclusion criteria. Nine articles concerned retinal cells, 5 concerned spinal cord injury, and 4 concerned amyotrophic lateral sclerosis. A multi-drug and short-term immunosuppression regime were commonly employed in the identified studies. Detected immune responses in treated patients were rare. Common immunosuppression paradigms included tacrolimus, mycophenolate mofetil and tapering doses of steroids. Local immunosuppression with steroids was employed in some studies concerning retinal diseases.

**Discussion:**

A short-term course of systemic immunosuppression seemed efficacious for most included studies, with some showing grafted cells viable months to years after immunosuppression had stopped. Longer-term follow-up is required to see if this remains the case. Side effects related to immunosuppression were uncommon.

## Introduction

Stem cells are unspecialised cells that have the potential to develop into many different cell types [1]. They have been intensively investigated in the field of regenerative medicine, where donor stem cells can be transplanted into the host to replace damaged cells. However, if the stem cells originate from a foreign source, there is potential for recognition by the host immune system. Here, immunosuppression can be used to dampen this response [2].

Immunosuppression has been used in stem cell trials on spinal cord injury, amyotrophic lateral sclerosis (ALS) and macular degeneration, among others [3–5]. Stem cell therapy for spinal cord injury has been trialled to potentially improve sensory and motor function following injury [3]. Similarly in ALS, stem cells have been employed to directly regenerate damaged nerve cells, or to create a neural population that provides a supportive environment for diseased motor neurones [5]. Within the retina, macular degeneration and Stargardt’s macular dystrophy are diseases that have been targeted by stem cell trials through replacement of the retinal pigment epithelium (RPE) [4,6]. The applicability of stem cell therapy is rapidly evolving to address neural deficits in similar organs and functions, such as the inner ear and hearing. To facilitate this translation, knowledge from investigation into similar organs and cellular targets must be appraised and adapted. This is particularly evident in the process of immunosuppression, where organ-specific pharmacodynamics and kinetics must be addressed, and the innate and adaptive organ-specific immune response modified.

Many different sources of stem cells have been used in regenerative studies, including human embryonic stem cells (hESCs), human umbilical-derived cells (hUDCs), and induced pluripotent stem cells (iPSCs). The ability of hESCs-derived RPE cells to be stored and readily available for any patient is an advantage [7]. However, as these cells are not derived from the patient, immunosuppression therapy is necessary to prevent rejection [8]. When allogenic stem cells are used for transplantation, immunosuppression is generally required to prevent immune rejection of these cells. However, immunosuppression is associated with a risk of side effects. These range from infection due to systemic immunosuppression, to agent-specific effects, such as diabetes mellitus, nausea, and diarrhoea [9,10]. The use of HLA-matched cells in these fields has been adopted in some studies to reduce the need for immunosuppression in the recipient, although debate remains on whether ongoing immune rejection of allogeneic transplanted stem cells occurs [11]. Long-term survival of stem cell grafts has been noted many months after immunosuppression has been stopped in some reports [12].

The objective of this systematic review is to analyse the literature of immunosuppression that has been used in stem cell trials concerning retinal and neural cells, which are cell types that have been targeted by most stem cell trials so far [13]. This will be useful in informing immunosuppression choice in future trials.

## Methods

**Fig 1:**
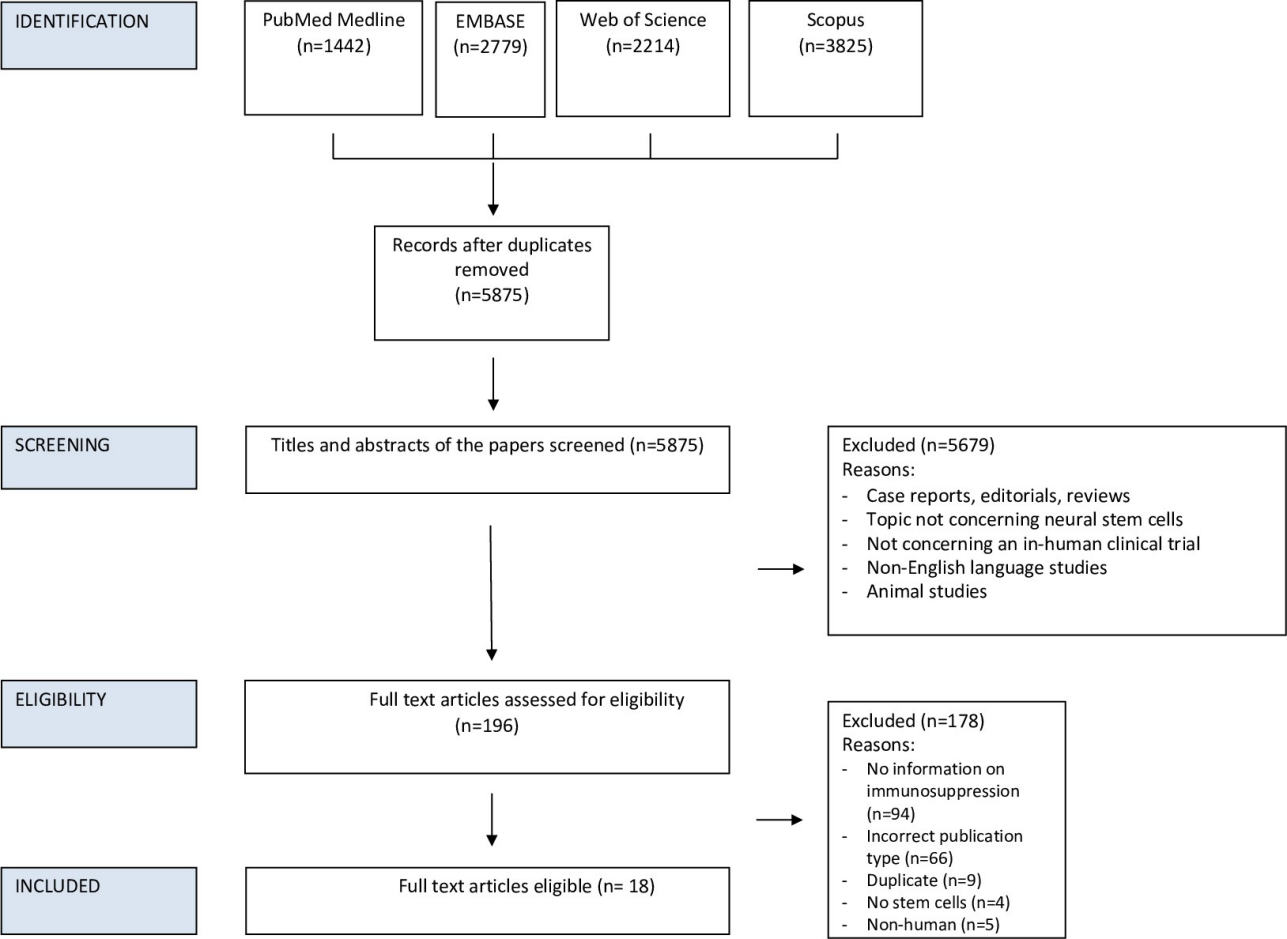
PRISMA flowchart outlining the screening process.

The databases Medline, Embase, Scopus, Web of Science, and the Cochrane Library were searched from inception to February 2024, individually. The review was not prospectively registered. Exact search terms used on each database (and each platform) are provided in Supplementary File 1. The search strategy was formulated by a medical librarian using the PRESS checklist and evaluated against the PRISMA-S guidelines [14,15]. Databases were searched separately, as opposed to multiple databases being searched simultaneously on the same platform. The search syntax was adapted for each database, to account for variation between thesaurus terms/controlled vocabulary across each database. Results were deduplicated using Endnote 20 software. Two authors (SG and DB) screened titles and abstracts generated by the search independently and then assessed the full texts of all relevant articles against the inclusion criteria (Fig 1). Any disagreement between the assessors on the suitability of articles for inclusion tackled by thorough discussion between assessors, or failing this, by referral to the senior author (MS). Studies were included in the review if they met the following criteria:

1. Presented data on neural or retinal cells as part of an in-human clinical trial
2. Detailed the immunosuppression regime used

Non-English studies were excluded, as were those not specifying if an immunosuppressive regimen was employed. Studies containing duplicated data from previous studies were excluded, along with animal studies, review articles, case reports, and editorials. There were no limits set on publication year. Data collection from included papers was performed in the same way as screening (i.e., independently by 2 reviewers). The ROBINS-I tool was used for risk of bias analysis for included studies [16]. Risk of bias analysis for each study was performed in the same manner as data extraction (i.e., independently by 2 authors, SG and DB). Due to the heterogeneity in presentation of data, a statistical meta-analysis of these results was not possible, and the results were presented descriptively in a table and focused on the below targeted outcomes.

The primary outcome of interest was the immunosuppression regimen used and associated evidence of immune rejection or graft cell differentiation and survival. The secondary outcome of interest was reported adverse events related to the use of the immunosuppression.

## Results

A literature search yielded 5875 papers after deduplication. Following screening of the title and abstract, 5679 articles were excluded on subject applicability, or identified as non-English language studies, non-human studies, editorials, case reports, reviews, or duplicated studies. The remaining 196 articles were reviewed by full text against the selection criteria. Of these, 178 were excluded (including 94 for not detailing immunosuppression used), leaving 18 articles for inclusion in this study [3–6,12,17–29]. This process is outlined as part of a PRISMA flowchart in Fig 1. Nine articles concerned retinal cells [4,6,17–20,27–29], 5 concerned spinal cord injury [3,23–26] and 4 concerned ALS [5,12,21,22]. The characteristics of these studies, summarising information provided on the immunosuppression used, are outlined in Tables 1–3.

**Table 1:** Study characteristics on stem cell trials concerning retinal diseases.

Paper and year	Phase of trial	Disease	Number of participants and mean age	Gender (male/female)	Type of stem cell	Type of immunosuppression	Dose of immunosuppression	Length of immunosuppression	Median follow-up	Stem cell rejection	Infectious adverse events	Need to stop the immunosuppressants
Song et al. 2015 [29]	I/IIa	Dry AMD (n = 2) Stargardt’s macular dystrophy (n = 2)	4 patients 57 years	4/0	Human embryonic-stem-cell (hESC)-derived retinal pigment epithelium	Tacrolimus MMF	Tacrolimus: titrated to achieve target blood concentrations 3–7 ng/mL MMF: 1.0–1.5 g/day	Tacrolimus: 1 week before surgery for 7 weeks MMF: 1 week before surgery for 14 weeks and then slowly tapered	12 months	No	1 pneumonia 2 upper respiratory tract infections 1 case of herpetic vesicles in the forearm	1 because of repeated deterioration of renal function and bone marrow suppression
Schwartz et al. 2016 [17]	I/II	Dry AMD (n = 9) Stargardt’s macular dystrophy (n = 9)	18 patients 63.5 years	7/11	Human embryonic-stem-cell (hESC)-derived retinal pigment epithelium	Tacrolimus MMF	Tacrolimus: titrated to achieve target blood concentrations 3–7 ng/mL MMF: 0.25–2.00 g/day	Tacrolimus: 1 week before surgery for 6 weeks MMF: 1 week before surgery for 12 weeks	22 months	No	1 urinary tract infection 1 culture- positive acute postoperative endophthalmitis (Staphylococcus epidermidis)	1 because of the urinary tract infection 1 to treat a post-operative infection (acute postoperative endophthalmitis
Liu et al. 2018[18]	I	Wet AMD	3 patients 63.7 years	1/2	Human embryonic-stem-cell (hESC)-derived retinal pigment epithelium	Tacrolimus MMF Prednisolone	Tacrolimus: s titrated to achieve target blood concentrations 10 ng/ml; 3–7 ng/ml at 1 month; 3 ng/ml at 2 months MMF: 200 mg (twice/day) Prednisone: 30 mg, once/day for 1 month, decreased to 15mg/day by the end of the 2 month, discontinued at 3months post op.	Tacrolimus: 1 week before surgery and discontinued at 4 months MMF: 1 week before surgery and discontinued by 2 months Prednisolone: 1 week before surgery and discontinued at 3 months	12 months	No	None	No
Da Cruz et al. 2018 [4]	I	Wet AMD	2 patients 72 years	1/1	Human embryonic-stem-cell (hESC)-derived retinal pigment epithelium	Prednisolone Triamcinolone acetonide Intraocular fluocinolone steroid implant	Prednisolone: 1 mg/kg oral prednisolone (up to 60 mg maximum) for at least 2 weeks and then tapered Triamcinolone acetonide: 40 mg Fluocinolone implant: either 0.19 or 0.59 mg	Prednisolone: 2–4 days before surgery—continued for at least 2 weeks followed by tapering over at least 1 further week Triamcinolone acetonide: 1 injection during surgery Fluocinolone implant: long-term intravitreal implant (time scale not specified)	12 months	A decrease in central pigmentation in patient 1 might have represented cell loss from delayed rejection	No	No
Mehat et al. 2018 [19]	I/II	Stargardt’s macular dystrophy	12 patients 44.4 years	11/1	Human embryonic-stem-cell (hESC)-derived retinal pigment epithelium	Tacrolimus MMF	Tacrolimus: titrated to achieve trough serum levels of 3 to 7 ng/ml MMF: escalating regimen: 0.25g twice daily (day 0 to day 1); 0.5 g twice daily (day 2 to day 3), and 1 g twice daily from day 4 until 12 weeks after transplantation, when it was discontinued	Tacrolimus: 1 week before surgery for 7 weeks MMF: commenced on the day of surgery for 12 weeks	12 months	No	1 herpes-simplex virus 1 reactivation	No
Sung et al. 2021 [20]	I	Stargardt’s macular dystrophy	3 patients 41.7 years	3/0	Human embryonic-stem-cell (hESC)-derived retinal pigment epithelium	Tacrolimus MMF	Tacrolimus: titrated to achieve a target through level of 3–7 ng/mL MMF: 1g/day orally	Tacrolimus and MMF: if clinically meaningful visual improvement was observed 3-months post-operatively, immunosuppression was continued and tapered down at approximately 1 year after transplantation	36 months	No	No	No
Li et al. 2021 [6]	I	Stargardt’s macular dystrophy	7 patients 23.2 years	2/5	Human embryonic-stem-cell (hESC)-derived retinal pigment epithelium	Tacrolimus MMF Prednisone	Tacrolimus: 0.1 mg/kg/day (target serum levels 3–7 ng/mL) MMF: 500mg twice a day Prednisolone 0.5 mg/kg/d initially	Tacrolimus: 1 week before surgery to 12 weeks after surgery MMF: 1 week before surgery to 4 weeks after surgery Prednisolone: started 1 week before surgery; decreased to 0.25 mg/kg/d at the 4th week and then stopped at the 12th week	60 months	No	No	No
Fernandes et al. 2023 [27]	I	Stargardt’s macular dystrophy	12 patients 41.5 years	3/9	Human embryonic-stem-cell (hESC)-derived retinal pigment epithelium	Prednisolone Cyclosporine	Prednisolone: 1 mg/kg/day of oral prednisone, with a dose decrease of 0.125 mg/kg/week Cyclosporine: 5 mg/kg/day	Prednisolone: Day 1 to Month 3 Cyclosporine: Day 1 to Month 3	12 months	No	No	No
Humayun et al. 2023 [28]	I/ IIa	Dry AMD	15 patients 77 years	6/9	Human embryonic-stem-cell (hESC)-derived retinal pigment epithelium	Tacrolimus Methylprednisolone sodium succinate	Tacrolimus: titrated to achieve target trough levels 3–10 ng/ml) Methylprednisolone sodium succinate: 250 mg	Tacrolimus: 8 days before surgery to 42 days after surgery. Tapering began at day 42 and was completed by day 60. Methylprednisolone: 1 dose 30 mins before surgery	36 months (median)	No	2 pneumonia 1 sepsis 1 COVID-19	1 due to pneumonia

Key: MMF = Mycophenolate mofetil; AMD = age-related macular degeneration.

**Table 2:** Study characteristics on stem cell trials concerning spinal cord injury.

Paper and year	Phase of trial	Number of participants and mean age	Gender (male/ female)	Type of stem cell	Type of immunosuppression	Dose of immunosuppression	Length of immunosuppression	Follow-up	Stem cell rejection	Infectious adverse events	Need to stop the immunosuppressants
Curtis et al. 2018 [23]	I	4 patients 30 years	3/1	Human spinal cord-derived neural stem cell (NSI-566)	Basiliximab Tacrolimus MMF	Basiliximab: 20 mg given within 2 hours prior to transplantation surgery and 20 mg on postoperative day 3 or 4 Tacrolimus: 0.1 mg/kg/day till week 12. Dose then halved for 1 week. Dose then halved again for another week, before being ceased. MMF: Day 1–7: 1000 mg/day Day 8–14: 1500 mg/day Day 15- week 12: 2000 mg/day. Dose then halved for 1 week. Dose then halved again for another week, before being ceased.	Basiliximab: Given on day of surgery and postoperative days 3 and 4 only. Tacrolimus: Started on post-transplant Day 1 and continued for 14 weeks. MMF: Started on post-transplant Day 1 and continued for 14 weeks.	18–27 months	No antibodies against the HLA alleles of the donor cells.	None	No
Levi et al. 2019 [24]	II	12 patients 28 years (median)	11/1	Human central nervous system stem cells (HuCNS-SC	Tacrolimus Trimethoprim/sulfamethoxazole MMF Dexamethasone	Tacrolimus: 0.2 to 0.3 mg/kg/day to target blood levels of 5–10 μg/L for first 28 days and 2–5 μg/L for the following 5 months. Trimethoprim/sulfamethoxazole: Not stated MMF: Not stated Dexamethasone: Not stated	Tacrolimus: Started 3 days pre-transplantation and continued for 6 months post-transplantation Trimethoprim/sulfamethoxazole: 6 months post-transplantation MMF: 1-month post-transplantation Dexamethasone: 1 day prior to and for 7 days post-transplantation.	9–12 months	Not stated	48 episodes of infection unspecified in the treated groups. 1 serious adverse event: staph epidermidis wound infection requiring incision and drainage and IV antibiotics	No
Curt et al. 2020 [3]	I/IIa	12 patients 31 years	11/1	Human central nervous system stem cells (HuCNS-SC	Tacrolimus MMF Dexamethasone	Tacrolimus: Not stated. Target blood levels were 5–10 ng/mL MMF: 500mg/day Dexamethasone: 8–16 mg/day	Tacrolimus: 9-month course started 3 days pre-transplantation MMF: 28-day course started 2 days pre-transplantation Dexamethasone: 5–10 days started 1 day pre-transplantation	72 months	Not stated	Subjects had an average of 1.1 UTIs in the first year after surgery and 0.66 UTIs over the next 5 years. 1 serious adverse event involving hospitalisation for a UTI	Not stated
McKenna et al. 2022 [25]	I	5 patients Age range: 21–32 years. Unable to calculate mean from data provided	4/1	Oligodendrocyte progenitor cells (LCTOPC1) derived from human pluripotent stem cells	Tacrolimus	Tacrolimus: 0.01 mg/kg/day IV. Oral tacrolimus was started as soon as possible at 0.03 mg/kg/day. Target levels were 3 to 7 ng/ml The tacrolimus dose was halved on day 46 and halved again on day 53. It was discontinued at Day 60	Tacrolimus: 60 days post-transplantation	120 months	No evidence of T-cell–mediated or antibody responses to LCTOPC1 even after cessation of tacrolimus immunosuppression	1 vaginal yeast infection and 6 UTIs	Not stated
Fessler et al. 2022 [26]	I/IIa	25 patients 31.8 years	21/4	Oligodendrocyte progenitor cells (LCTOPC)	Tacrolimus	Tacrolimus: Starting dose = 0.01 mg/kg/day Levels were titrated to target levels 3–7 ng/ml	Tacrolimus: Started 6–12 hours before injection of stem cells and continued for 60 days post-transplantation Dose was halved on Day 46 and halved again on Day 53 before being stopped on Day 60	12 months	Not stated	1 case of bacterial infection (unspecified) as a serious adverse event thought to be related to tacrolimus and resolved with antibiotics	Not stated if tacrolimus was stopped in the bacterial infection case

Key: MMF = Mycophenolate mofetil.

**Table 3:** Study characteristics on stem cell trials concerning Amyotrophic lateral sclerosis (ALS).

Paper and year	Phase of trial	Number of participants and mean age	Gender (male/ female)	Type of stem cell	Type of immunosuppression	Dose of immunosuppression	Length of immunosuppression	Follow-up	Stem cell rejection	Infectious adverse events	Need to stop the immunosuppressants
Glass et al. 2012 [22]	I	12 patients 52 years	12/0	Human spinal cord–derived neural stem cells	Basiliximab Prednisone Tacrolimus MMF	Basiliximab: 20mg Prednisone: 60mg Tacrolimus: Not stated, but dosed to maintain target levels of 4–8 ng/ml MMF: 1000mg	Basiliximab: 20mg during surgery and on postoperative day 4 Prednisone: Tapered to 0mg over 1 month Tacrolimus: continuous for duration of trial MMF: continuous for duration of trial	6–18 months	Immunologic studies did not show markers of rejection of the transplanted cells	No opportunistic infections. 2 SAEs involving pneumonia	Tacrolimus: 3 patients stopped taking and dose reduced in 2 due to vomiting and diarrhoea MMF: 2 patients stopped taking and dose reduced in 2 (unspecified reason)
Glass et al. 2016 [5]	I/II	15 patients 48 years	12/3	Human spinal cord–derived neural stem cells	Basiliximab Prednisone Tacrolimus MMF	Basiliximab: 20mg Prednisone: 60mg Tacrolimus: Not stated, but dosed to maintain target levels of 4–8 ng/ml. MMF: 1000mg	Basiliximab: 20mg during surgery and on postoperative day 4 Prednisone: Tapered to 0mg over 1 month Tacrolimus: continuous for duration of trial MMF: continuous for duration of trial	24 months and then at every 6 months until death	Immunologic studies did not show markers of rejection of the transplanted cells	9 adverse events related to infection (3 pneumonia; 3 UTI; 3 unspecified)	Tacrolimus: 4 stopped due to new-onset diabetes mellitus (2), headache (1) and diarrhoea (1) MMF: 2 stopped due to headache (1) and diarrhoea (2)
Mazzini et al. 2019 [21]	I	18 patients 48 years (median)	13/5	Human neural stem cells	Tacrolimus Methylprednisolone Prednisone	Tacrolimus: 0.2 mg/kg/day. Target concentrations were 5–10 ng/ml Methylprednisolone: 125 mg Prednisone: 60mg tapered to 0mg over 1 month	Tacrolimus: 6 months Methylprednisolone: Once only 2 hours before surgery Prednisone: 1 month	24 months (median)	Not stated	2 patients developed pneumonia	Tacrolimus was suspended prematurely in 1 patient due to postural tremor
Baloh et al. 2022 [12]	I/IIa	18 patients 58 years	8/10	Human neural progenitor cells transduced with GDNF (CNS10-NPC-GDNF)	Basiliximab Tacrolimus MMF Methylprednisolone Prednisone	Basiliximab: 20 mg Tacrolimus: 1–6 mg/day MMF: 1g/day Methylprednisolone: 125 mg Prednisone: 60mg tapered to 0mg over 1 month	Basiliximab: 1 dose intraoperatively and on post-op day 4 Tacrolimus: 12 months MMF: Not stated Methylprednisolone: Once intraoperatively Prednisone: 1 month	12 months	Donor-specific antibodies were detected only in 1 participant who had widespread inflammatory reaction to transplanted cells	4 patients developed UTI 1 patient developed Clostridium Difficile colitis	Tacrolimus was suspended prematurely in 2 patients due to adverse events unspecified

Key: MMF = Mycophenolate mofetil.

### Stem cell trials concerning retinal diseases

Transplantation of human embryonic stem cell (hESC)-derived retinal pigment epithelial (RPE) cells offers the potential for benefit in macular degeneration, with reports of improved visual acuity [19].

The characteristics of hESC RPE cell trials for the treatment of macular degeneration, including the types, doses, and length of immunosuppression, are outlined in Table 1. Systemic immunosuppression generally involved use the combination of tacrolimus and mycophenolate mofetil (MMF) in 6/9 reports. The use of steroids was found in 5/9 papers. In only 1 study was steroid-based local immunosuppression used [4], and subjects received an intravitreal implant of fluocinolone acetonide (either 0.19mg or 0.59mg) as an anti-inflammatory and immunosuppressive agent.

The duration of immunosuppression varied depending on the drug used. Mycophenolate mofetil was typically used for a longer-period of time, with doses ranging from 0.2–2.00 g orally per day; Tacrolimus was adjusted to a target serum concentration (Table 1).

All studies utilised cells derived from human embryonic stem cells. Signs of immune rejection were defined as including vitritis, retinitis, retinal exudates, retinal oedema, or vascular hyperpermeability. None of the studies described clear features of rejection of the transplanted cells, with no detection of intraretinal fluid on optical coherence tomography (OCT), and no changes on fundus fluorescein angiography to suggest rejection. 1 study reported a decrease in central pigmentation which could represent cell loss from a delayed rejection, but normal architecture and retinal function were maintained [4].

Serious adverse events were rare, including infectious adverse events (Table 1). The most common adverse events were gastro-intestinal symptoms, infections (urinary tract infections, pneumonia, herpes simplex virus reactivation), headache, lethargy, nausea. 1 study reported patients developing non-melanoma skin cancers [17], and 1 study reported a patient developing an upper-GI cancer [28], with malignancies deemed unlikely to be related to the immunosuppressants.

### Stem cell trials concerning spinal cord injury

Five studies were identified as reporting human clinical stem cell trials for spinal cord injury. The characteristics of the trials, including the types, doses, and length of immunosuppression, are outlined in Table 2. Systemic immunosuppression including use of the calcineurin inhibitor tacrolimus was employed in all 5 studies. Immunosuppression was achieved with multiple agents in 3 of the 5 studies, including combinations of mycophenolate mofetil, basiliximab, or dexamethasone (Table 2). No studies used a local route of immunosuppressant administration. The duration of immunosuppression varied depending on the drug used. Tacrolimus was typically used for a longer-period of time, with doses adjusted to target serum concentrations, while other drugs such as dexamethasone and basiliximab were used around the perioperative period only (Table 2).

Human central nervous system stem cells were transplanted in 3 of 5 papers [3,23,24]. In 2 studies, oligodendrocyte progenitor cells derived from human pluripotent stem cells were used [25,26]. Variable signs of an immune response or rejection were sought, including the development of antibodies against the HLA antigens of the donor cells. No evidence of rejection was reported in any study. Serious adverse events that may be attributable to immunosuppression were rare, but included cases of hospitalisation for UTI and bacterial infection [26]. Patients with spinal cord injury are already susceptible to UTIs due to urinary statis secondary to neurogenic bladder [30], and 1 study found that the number of UTIs per year in immunosuppressed trial participants was similar to prior estimates in the general spinal cord injury population [3,30].

### Stem cell trials concerning ALS

Several studies have explored the use of stem cells as a treatment for amyotrophic lateral sclerosis. Transplanted stem cells secrete neurotrophic factors, differentiate into supporting cells such as astrocytes and microglia, and delay the degeneration of motor neurons [31].

The characteristics of stem cell trials on ALS, including the types, doses, and length of immunosuppression, are outlined in Table 3. All 4 identified studies utilised systemic immunosuppression, involving tacrolimus and additional agents, including mycophenolate mofetil in 3 of 4 studies. Tacrolimus was typically continued throughout the course of the trial and doses were adjusted to allow a target serum level between 4–10 ng/ml. Mycophenolate was taken regularly at variable doses ranging from 125-1000mg a day. Some immunosuppressants were restricted to the perioperative period only, and included basiliximab (a chimeric anti-intcrleukin-2 receptor monoclonal antibody) and methylprednisolone. Prednisolone was also used in the postoperative period, with doses typically being tapered down to 0 mg by 1 month. Monitoring for signs of an immune response against donor stem cells involved testing for the formation of host antibodies against these cells. This was rare, with only 1 study describing formation of these antibodies along with an inflammatory reaction around the transplanted cells [12]. In this study, 2 donor specific antibodies were positive at baseline testing, and increased in specificity throughout the study, and there was 1 case of *de novo* donor specific antibody. The study did not state when the latter was first detected, and while there was immune reaction, there was still graft survival in this case on autopsy.

Infectious adverse events typically included respiratory or urinary tract infections, but these were rare (Table 3). ALS patients are susceptible to pneumonia due to decreased respiratory function, weakened respiratory musculature, and increased risk of aspiration [32]. As such, it is unclear the extent to which immunosuppression contributed to these cases. Patients typically tolerated immunosuppression well and these medications were only rarely stopped. Tacrolimus and mycophenolate were mainly stopped due to gastrointestinal side effects such as nausea and vomiting, although this was rare (Table 3).

The central nervous system is thought to be an immune-privileged site. In the ALS trials involving allogenic stem cells, immunosuppression was temporary and lasted for the duration of the trial. However, participants were typically followed-up after this time when immunosuppression was stopped. In these cases, there was graft survival confirmed many months after immunosuppression was ceased, suggesting that continuous immunosuppression might not be necessary, potentially reducing the risk of long-term adverse events.

### Quality assessment

The quality of included studies was assessed using the ROBINS-I tool and is included in Table 4. Nine studies were classified as being at moderate risk of bias, and 9 studies were classified as being low risk of bias.

**Table 4:** Risk of Bias analysis of included studies using the ROBINS-I quality assessment tool.

Study Name	Bias due to confounding	Bias in the selection of participants in the study	Bias in the classification of interventions	Bias due to deviations from intended interventions	Bias due to missing data	Bias in the measurement of outcomes	Bias in selection of the reported result	Overall
Glass et al. 2012 [22]	N/A	Moderate	Low	Low	Low	Low	Low	Low
Song et al. 2015 [29]	Moderate	Moderate	Low	Low	Low	Low	Low	Moderate
Glass et al. 2016 [5]	N/A	Moderate	Low	Low	Low	Low	Low	Low
Schwartz et al. 2016 [17]	Moderate	Moderate	Low	Low	Low	Low	Low	Moderate
Liu et al. 2018 [18]	Moderate	Moderate	Low	Low	Low	Low	Low	Moderate
Da Cruz et al. 2018 [4]	Moderate	High	Low	Low	Low	Low	Low	Moderate
Mehat et al. 2018 [19]	Moderate	Moderate	Low	Low	Low	Low	Low	Moderate
Curtis et al. 2018 [23]	N/A	Moderate	Low	Low	Low	Low	Low	Low
Levi et al. 2018 [24]	N/A	Moderate	Low	Low	Low	Low	Low	Low
Mazzini et al. 2019 [21]	N/A	High	Low	Low	Low	Low	Low	Low
Curt et al. 2020 [3]	N/A	Moderate	Low	Low	Low	Low	Low	Low
Li et al. 2021 [6]	Moderate	Moderate	Low	Low	Low	Low	Low	Moderate
Sung et al. 2021 [20]	Moderate	Moderate	Low	Low	Low	Low	Low	Moderate
Baloh et al. 2022 [12]	N/A	Moderate	Low	Low	Low	Low	Low	Low
McKenna et al. 2022 [25]	N/A	Moderate	Low	Low	Low	Low	Low	Low
Fessler et al. 2022 [26]	N/A	Moderate	Low	Low	Low	Low	Low	Low
Fernandes et al. 2023 [27]	Moderate	Moderate	Low	Low	Low	Low	Low	Moderate
Humayun et al. 2023 [28]	Moderate	Moderate	Low	Low	Low	Low	Low	Moderate

## Discussion

To inhibit potential immune response and inflammation following stem cell grafting, all studies balanced the benefit of immunosuppression with the potential side effects related to this treatment.

The long-term viability of stem cell derived RPE cells transplanted in the subretinal space, and the role of immunosuppression in cell survival in clinical trials remains unclear. Animal model studies in recent years have added to our understanding of the role of immunosuppression in localised neural stem cell transplants. A series of pre-clinical studies completed by da Cruz *et al*. suggests a role for immunosuppression [4]. In a porcine-model study where no immunosuppression was used, no definitive hESC RPE cells were identified at 26 weeks, whereas persistent hESC RPE cells on histology were found at 6 weeks in the earlier similar porcine studies where animals were immunosuppressed perioperatively. The group also identified viable RPE cells at 26 weeks in a study utilising immune-deficient mice [4].

McGill *et al*. transplanted induced pluripotent stem cells derived RPE cells into the subretinal space of non-immunosuppressed rhesus monkeys, demonstrating that the cells were no longer detectable 3 weeks after transplantation due to rejection by the immune system [33]. Szatmári-Tóth et al [34] reported that dying hESC-RPEs are efficiently engulfed by macrophages, resulting in the release of high amounts of IL-6 and IL-8 cytokines. In our review, 1 study reported a decrease in central pigmentation in 1 patient 12 months post-transplantation that could have represented cell loss from delayed rejection (Table 1). However, the remaining studies did not report signs of rejection in the time frame studied. Although adverse events related to the immunosuppressive medications occurred during the period of their administration, serious adverse events were rare (Table 1).

Additionally, mesenchymal and hESC-derived RPE cells have also been shown to harbour intrinsic immunomodulatory properties [35,36]. In vitro experiments have demonstrated that they can inhibit the proliferation of activated T cells, reduce the secretion of pro-inflammatory cytokines such as interferon-γ, and enhance T cell apoptosis [36,37]. In vivo experiments in rodents have also shown that they can increase the expression of anti-inflammatory cytokines such as IL-10 [36].

In the ALS trials described involving allogenic stem cells (Table 3), immunosuppression was temporary and lasted only for the duration of the trial. However, participants were typically followed-up after this time when immunosuppression was stopped. In these cases, there was graft survival many months after immunosuppression was ceased, suggesting that continuous immunosuppression might not be necessary. This can be beneficial for patients as a reduced course of immunosuppression reduces the risk of experiencing side effects during the course.

Most studies used a short course of immunosuppression around the transplantation period, with most finding little evidence of immune rejection (Tables 1–3). Proposed mechanisms to explain the sufficiency of a short course include the immune-privileged nature of the central nervous system and retina, and the intrinsic immunomodulatory properties of stem cells that can dampen the local immune response [12,36,38]. However, most studies had a follow-up period of less than 2 years (Tables 1–3), and it is possible that rejection can occur after this period. If this is the case, it might necessitate the consideration of long-term immunosuppression, as is currently the case for organ transplantation. However, there is insufficient long-term data available from these studies (Tables 1–3) to comment on the need for long-term immunosuppression.

### Suppression of stem cell graft rejection in animal models with multiple agents

Several animal studies have shown that transplantation of allogeneic stem cells can result in immune cell infiltration of the transplanted tissue and subsequent rejection in immunocompetent models [39,40]. In a xenograft model in 2008, Swijnenburg *et al*. characterised the role single and multiple agent immunosuppression may play in hESCs transplants. Tracking the fate of transplanted stem cells via bioluminescent imaging [41], they showed that hESC survival after transplantation was shorter in immunocompetent mice compared to immunodeficient mice. In addition, they demonstrated that using immunosuppressive drugs prolonged cell survival: compared to a control untreated group, tacrolimus-only and sirolimus-only treated groups displayed significantly longer stem cell survival, up to 5- and 7-days post-transplant respectively. However, for both groups, bioluminescent signals had decreased to background levels by day 10 post-transplant, highlighting the strong anti-hESC immune response. The use of mycophenolate mofetil did not result in a significant increase in hESC survival compared to using tacrolimus or sirolimus monotherapy. However, using a combination immunosuppressive regimen with tacrolimus and sirolimus extended hESC survival most when compared to the non-treated group, with hESC survival up to 28 days post-transplant. These immunosuppressants have distinct mechanisms of action, and by affecting different molecular pathways, a synergistic effect could occur [42]. Mycophenolate mofetil dampens the T and B cell immune response by inhibiting inosine monophosphate dehydrogenase, an enzyme that is required in the synthesis of guanosine nucleotides. It also promotes apoptosis of activated T lymphocytes [43]. Tacrolimus inhibits gene expression of IL2 –a pro-inflammatory cytokine–by inhibiting calcineurin phosphatase [44]. In contrast, sirolimus interrupts the signal from the activated IL2 receptor by inhibiting an enzyme called mTOR, thus suppressing IL2-driven T cell proliferation [45].

In a mouse model with implanted mouse ESCs, Pearl et al [46] showed that the use of costimulatory receptor blocking antibodies (CTLA4-Ig, anti-LFA-1, anti-CD40L) administered for a short duration (up to day 6 post-transplantation), combined with dual therapy with tacrolimus and sirolimus, prevented mESC rejection up to 28 days post transplantation. In contrast, the combined use of tacrolimus and sirolimus only, led to significantly reduced mESC survival at day 28 compared to the above regimen.

### Autologous stem cell transplantation

A way of dampening the host immune response is generally required when foreign cells are transplanted into a host. However, if the stem cells transplanted come from the host (autologous stem cells), immunosuppression is not required. Recent advances in stem cell research have led to the creation of induced pluripotent stem cells (iPSCs) [47]. These stem cells can be reprogrammed from differentiated adult cells, such as fibroblasts, through introducing growth factors. They have been used in animal models, including for Parkinson’s disease. Data in Parkinsonian primate models has shown long-term survival of implanted autologous iPSC-derived dopamine neurones and functional improvements in symptoms [48].

### Transplantation of early foetal cells

This review focused on trials transplanting stem cells directly into patients. Other groups have investigated transplanting early developmental foetal cells, such as foetal ventral mesencephalic cells (fVMs) [49] instead. These cells have been used in Parkinson disease models where they have shown long-term survival and improvements in disease symptoms. Human pluripotent stem cells (hESCs or iPSCs) can be differentiated into midbrain dopaminergic neurones [50,51]. If the source of these cells come from a foreign donor, immunosuppression will generally be indicated. The immunogenicity of dopaminergic neurones derived from such stem cells is not yet clear. It is thought that a period of immunosuppression will be required (1–2 years) following grafting, with a multi-modal immunosuppressive regime leading to better graft dopaminergic neuron survival. This is based on prior studies using fVMs [49].

In our review, all studies were Phase I/IIa studies, and most were scored as having a low or moderate risk of bias using the ROBINS-I quality assessment tool (Table 4).

### Limitations

The review was not prospectively registered on PROSPERO. The grey literature was also not systematically searched to identify studies outside the purview of the central databases searched and the review process would have missed trials currently in progress with preliminary data, but our focus was on published peer-reviewed work. The review also focused on neural and human embryonic stem cells, and as a result, did not include other types, such as bone marrow-derived mesenchymal stem cells among the sources of stem cells.

## Conclusions

To inhibit potential immune response and inflammation, all the studies included immunosuppression, with most opting for a multi-drug immunosuppression regime (Tables 1–3). Detected immune responses in treated patients were rare [12]. Immunosuppression for stem cell trials concerning spinal cord injury and ALS generally involve systemic immunosuppression involving tacrolimus, mycophenolate mofetil and tapering doses of steroids. These systemic immunosuppressants have also been used for trials concerning retinal diseases, although here local immunosuppressants with steroids have also been trialled. The central nervous system and the retina are immune-privileged sites, reducing the need for high doses of immunosuppression. These trials have generally shown that stem cell grafts remain viable months to years after immunosuppression has stopped. Side effects related to immunosuppression, including systemic infection, or those related to medications have been reported, even if uncommon. Adverse events related to the immunosuppressive medications occurred during the period of their administration, but serious adverse events were rare.

These findings suggest possible immunosuppression paradigms with stablished efficacy that may be translated to similar organs and neural microenvironments for future novel therapies, such as those concerning inner ear cells. The application of these paradigms will require further research towards target organ specific pharmacokinetics and dynamics, as well as organ specific adverse events. The findings detailed in this review present a foundation for the commencement of novel organs specific stem cell therapies.

## Supporting information

S1 Checklist PRISMA 2020 checklist. (DOCX)

S1 File Search strategy for the systematic review. (DOCX)
