# Supplementary material for: Immunosuppression in stem cell clinical trials of neural and retinal cell types: A systematic review
Source: PLoS One. 2024 Jul 5;19(7):e0304073. doi: 10.1371/journal.pone.0304073 (PMC11226136; doi:10.1371/journal.pone.0304073)
Supplement: S1 File — (DOCX) [file pone.0304073.s002.docx]

**Methods: Search Strategy**

The databases Medline (via Ovid), Embase (via Ovid), Web of Science (Core Collection) and Scopus were searched from inception to February 2024 by Veronica Phillips (Medical Librarian). The search strategy was peer-reviewed by two librarian colleagues of Veronica Phillips using the Peer Review of Electronic Search Strategies (PRESS) checklist (McGowan et al., 2016), and evaluated against the PRISMA-S guidelines (Rethlefsen et al., 2021). The completed PRISMA-S checklist is shown in the supplementary material. Databases were searched by the medical librarian separately, rather than multiple databases being searched on the same platform. The search syntax was adapted for each database, and to account for variation between thesaurus terms/controlled vocabulary across each database. No limits were applied to the searches. Results were imported to Endnote 20 by the medical librarian for deduplication, using the method outlined by Bramer et al. (Bramer et al., 2016). The searches will all be rerun prior to submission in order to include any papers published between the initial searching, and submission for peer review. Dates when searches were run will be indicated in the results table. The search strategies used in each database are documented in full below.

**Ovid MEDLINE(R) and Epub Ahead of Print, In-Process, In-Data-Review & Other Non-Indexed Citations, Daily and Versions 1946 to February 09, 2024**

(RPE or "retinal pigment epithelium" or neurodegen* or Parkinson* or Alzheimer* or Huntington or ALS or "Amyotropic lateral sclerosis" or "Motor neuron disease" or MND or spinal).ti,ab,kw. OR retinal pigment epithelium/ OR neurodegenerative diseases/ OR Parkinson Disease/ OR Alzheimer Disease/ OR Huntington Disease/ OR Amyotropic lateral sclerosis/ OR Motor Neuron Disease/

AND

("stem cell*" OR "precursor cell*").ti,ab,kw. OR Stem Cells/

AND

("clinical trial*" OR "randomized controlled trial*" or "randomised controlled trial*" or RCT* OR "phase I" OR "phase II" OR "phase III").ti,ab,kw. OR Randomized Controlled Trials as Topic/ OR randomized controlled trial/ OR clinical trial/ OR exp Clinical Trials as topic/

**Embase 1974 to 2024 February 09**

(RPE or "retinal pigment epithelium" or neurodegen* or Parkinson* or Alzheimer* or Huntington or ALS or "Amyotropic lateral sclerosis" or "Motor neuron disease" or MND or spinal).ti,ab,kw. OR retinal pigment epithelium/ OR neurodegenerative diseases/ OR Parkinson Disease/ OR Alzheimer Disease/ OR Huntington Disease/ OR Amyotropic lateral sclerosis/ OR Motor Neuron Disease/

AND

("stem cell*" OR "precursor cell*").ti,ab,kw. OR Stem Cell/

AND

("clinical trial*" OR "randomized controlled trial*" or "randomised controlled trial*" or RCT* OR "phase I" OR "phase II" OR "phase III").ti,ab,kw. OR "randomized controlled trial (topic)"/OR randomized controlled trial/ OR exp clinical trial/ OR "Clinical Trials (topic)"/

**Web of Science (Core Collection)**

TS=(RPE or "retinal pigment epithelium" or neurodegen* or Parkinson* or Alzheimer* or Huntington or ALS or "Amyotropic lateral sclerosis" or "Motor neuron disease" or MND or spinal)

AND

TS=("stem cell*" OR "precursor cell*")

AND

TS=("clinical trial*" OR "randomized controlled trial*" or "randomised controlled trial*" or RCT* OR "phase I" OR "phase II" OR "phase III")

**Scopus**

Title-Abs-Key (RPE or "retinal pigment epithelium" or neurodegen* or Parkinson* or Alzheimer* or Huntington or ALS or "Amyotropic lateral sclerosis" or "Motor neuron disease" or MND or spinal)

AND

Title-Abs-Key ("stem cell*" OR "precursor cell*")

AND

Title-Abs-Key ("clinical trial*" OR "randomized controlled trial*" or "randomised controlled trial*" or RCT* OR "phase I" OR "phase II" OR "phase III")

**Results Table**

| Database | Number of articles |
| --- | --- |
| Medline | 1442 |
| Embase | 2779 |
| Web of Science | 2214 |
| Scopus | 3825 |

Results after deduplication: 5875

**Reference List**

BRAMER, W. M., GIUSTINI, D., DE JONGE, G. B., HOLLAND, L. & BEKHUIS, T. 2016. De-duplication of database search results for systematic reviews in EndNote. *J Med Libr Assoc,* 104**,** 240-3.

MCGOWAN, J., SAMPSON, M., SALZWEDEL, D. M., COGO, E., FOERSTER, V. & LEFEBVRE, C. 2016. PRESS peer review of electronic search strategies: 2015 guideline statement. *Journal of clinical epidemiology,* 75**,** 40-46.

RETHLEFSEN, M. L., KIRTLEY, S., WAFFENSCHMIDT, S., AYALA, A. P., MOHER, D., PAGE, M. J. & KOFFEL, J. B. 2021. PRISMA-S: an extension to the PRISMA statement for reporting literature searches in systematic reviews. *Systematic reviews,* 10**,** 1-19.
